# A Novel Insecticidal Peptide SLP1 Produced by *Streptomyces laindensis* H008 against *Lipaphis erysimi*

**DOI:** 10.3390/molecules21081101

**Published:** 2016-08-22

**Authors:** Lijian Xu, Kangkang Liang, Bensha Duan, Mengdi Yu, Wei Meng, Qinggui Wang, Qiong Yu

**Affiliations:** 1 Key Laboratory of Microbiology, College of Life Science, Heilongjiang University, Harbin 150080, Heilongjiang, China; xulijian@hlju.edu.cn (L.X.); 2141174@s.hlju.edu.cn (K.L.); 2141169@s.hlju.edu.cn (B.D.); ymengdi@yahoo.com (M.Y.); 2 College of Agricultural Resource and Environment, Heilongjiang University, Harbin 150080, China; qgwang1970@163.com; 3 College of Life Science, Northeast Forestry University, Harbin 150040, China; mengwei@nefu.edu.cn

**Keywords:** actinobacteria, *Streptomyces*, peptide, insecticidal, *Lipaphis erysimi*, *Rhopalosiphum pseudobrassicae*, pesticide, aphid

## Abstract

Aphids are major insect pests for crops, causing damage by direct feeding and transmission of plant diseases. This paper was completed to discover and characterize a novel insecticidal metabolite against aphids from soil actinobacteria. An insecticidal activity assay was used to screen 180 bacterial strains from soil samples against mustard aphid, *Lipaphis erysimi*. The bacterial strain H008 showed the strongest activity, and it was identified by the phylogenetic analysis of the 16S rRNA gene and physiological traits as a novel species of genus *Streptomyces* (named *S. laindensis* H008). With the bioassay-guided method, the insecticidal extract from *S. laindensis* H008 was subjected to chromatographic separations. Finally, a novel insecticidal peptide was purified from *Streptomyces laindensis* H008 against *L. erysimi*, and it was determined to be S-E-P-A-Q-I-V-I-V-D-G-V-D-Y-W by TOF-MS and amino acid analysis.

## 1. Introduction

Actinobacteria are considered as important reservoir of antibiotics and pesticides [1,2]. *Streptomyces* species as actinobacteria are able to produce abundant metabolites with the insecticidal, antimicrobial, anti-tumor, and immunosuppressant activities [3,4]. Aphids are exclusive phloem feeders of the great majority of important plants in agriculture, horticulture and forestry, causing damage by direct feeding and transmission of plant diseases [5]. Mustard aphid (also known as Turnip aphid), *Lipaphis erysimi* (*Rhopalosiphum pseudobrassicae*), is a major insect pest attacking many closely related vegetables, including mustard, broccoli, cabbage, collards and radish [6]. Peptides are a valuable alternative to chemical pesticides for control of insect pests in agriculture because of their high kill efficiency, pest-specificity and their safety for the environment [7,8]. Recently, the peptides that are from Actinobacteria or other microorganisms exhibited their insecticidal potential [9,10]. However, the insecticidal peptide from Actinobacteria against *L. erysimi* has never been found before as far as we know. This study was to discover a new actinobacteria that can produce a new insecticidal peptide against *L. erysimi*. Therefore, we isolated many actinobacteria from the soil and investigated their insecticidal activity. Of them, the strain H008 showed a good insecticidal activity against *L. erysimi*, and *S. laindensis* H008 was identified as a novel species. Finally, we purified and characterized a novel insecticidal peptide SLP1 (means *Streptomyces laindensis* H008’s peptide No. 1) from *S. laindensis* H008 against *L. erysimi*.

## 2. Results

### 2.1. Microorganisms

A total of 180 bacterial strains were obtained from the soil samples. The strain H008 exhibited the highest insecticidal activity. The strain H008 was identified by its morphological and physiological traits and molecular biological methods.

#### 2.1.1. Morphological and Physiological Traits of Strain H008

**Figure 1:**
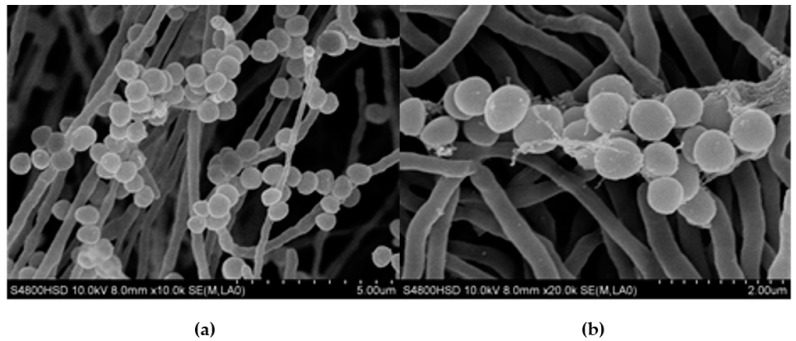
Morphology of the strain H008: (**a**) scanning electron microscope images of strain H008 (12,000×); and (**b**) scanning electron microscope images of strain H008 (30,000×).

The cultural characteristics of strain H008 are presented in Table 1. The strain H008 belongs to a spiral type, and its sporophore morphology was shown in Figure 1. The physiological and biochemical characteristics of strain H008 was shown in Table 2. The strain H008 utilized d-fructose, d-galactose, d-glucose, d-xylose, d-mannitol and sucrose as carbon sources, indicating its wide pattern of carbon assimilation. It exhibited salt tolerance up to 7% that may be placed in the intermediate group of salt tolerance. The melanin reaction was positive on ISP-6 medium. It was also found positive for biochemical tests like H_2_S, liquefaction of gelatin, starch hydrolysis, nitrate reduction and citrate utilization. Based on its morphological and physiological traits (Table 1 and Table 2, Figure 1), the strain H008 was a new species of the genus *Streptomyces* [11,12,13,14,15].

#### 2.1.2. Molecular Biological Identification of Strain H008

**Figure 2:**
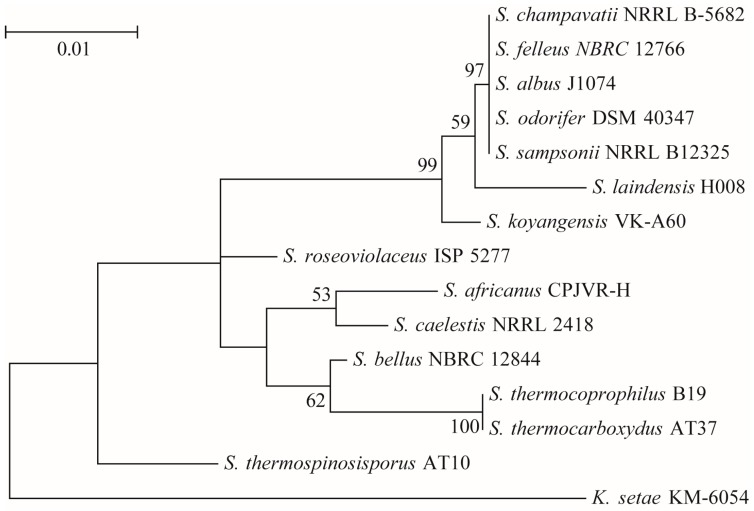
The phylogenetic analysis of strain H008. The sequences used in the analysis were obtained from the GenBank Database. The numbers at the branch nodes are the percentage bootstrap supports based on 1000 resample data sets. The scale bar corresponds to 0.01 substitutions per nucleotide position.

The 16S rDNA sequence of *Streptomyces* sp. H008 was amplified by the general primers for the *Streptomyces* species. The PCR yielded 1.053 kb 16S rDNA of *Streptomyces* sp. H008. The GenBank Accession number of *Streptomyces* sp. H008 was KX002028. The 16S rDNA sequence of *Streptomyces* sp. H008 was investigated by a similarity analysis (BLAST: https://blast.ncbi.nlm.nih.gov/Blast.cgi) and a phylogenetic analysis (Figure 2). The Hasegawa-Kishino-Yano model was selected to construct the Maximum Likelihood (ML) tree by finding the best model for ML tree after the alignment. All of these results indicated that strain H008 was a new species of genus *Streptomyces* that was named *S. laindensis* H008.

### 2.2. Insecticidal Activity

To investigate the stability of the insecticidal activity against *L. erysimi*, the culture filtrates of the *S. laindensis* H008 were stored at different pH values (from 2.0 to 11.0) for 24 h, or they were treated with different temperatures (20 to 80 °C) for 1 h. After storage at pH 2.0 to 11.0 for 24 h, the insecticidal activity of the culture filtrate was slightly (but significantly) changed and the mortality was 81.73% (the lowest one at pH 11.0) to 88.81% (the highest one at pH 6.5) by using one-way ANOVA with the Fisher least significant difference (LSD) post hoc test (*p* < 0.05). In addition, the mortalities of the culture filtrates against *L. erysimi* at 20, 30, 40, 50, 60, 70 and 80 °C were 88.82%, 85.65%, 82.57%, 78.74%, 75.86%, 73.32% and 70.11%, respectively. During the purification, each fraction was investigated its insecticidal activity and the result was shown in Table 3. In two steps of purification, the fraction No. 1B_12_-1C_1_ in the first step and the fraction No. 2D_1_-2D_2_ in the second step showed the highest activity, respectively.

### 2.3. Purification and Identification of Insecticidal Peptide

#### 2.3.1. Purification

**Figure 3:**
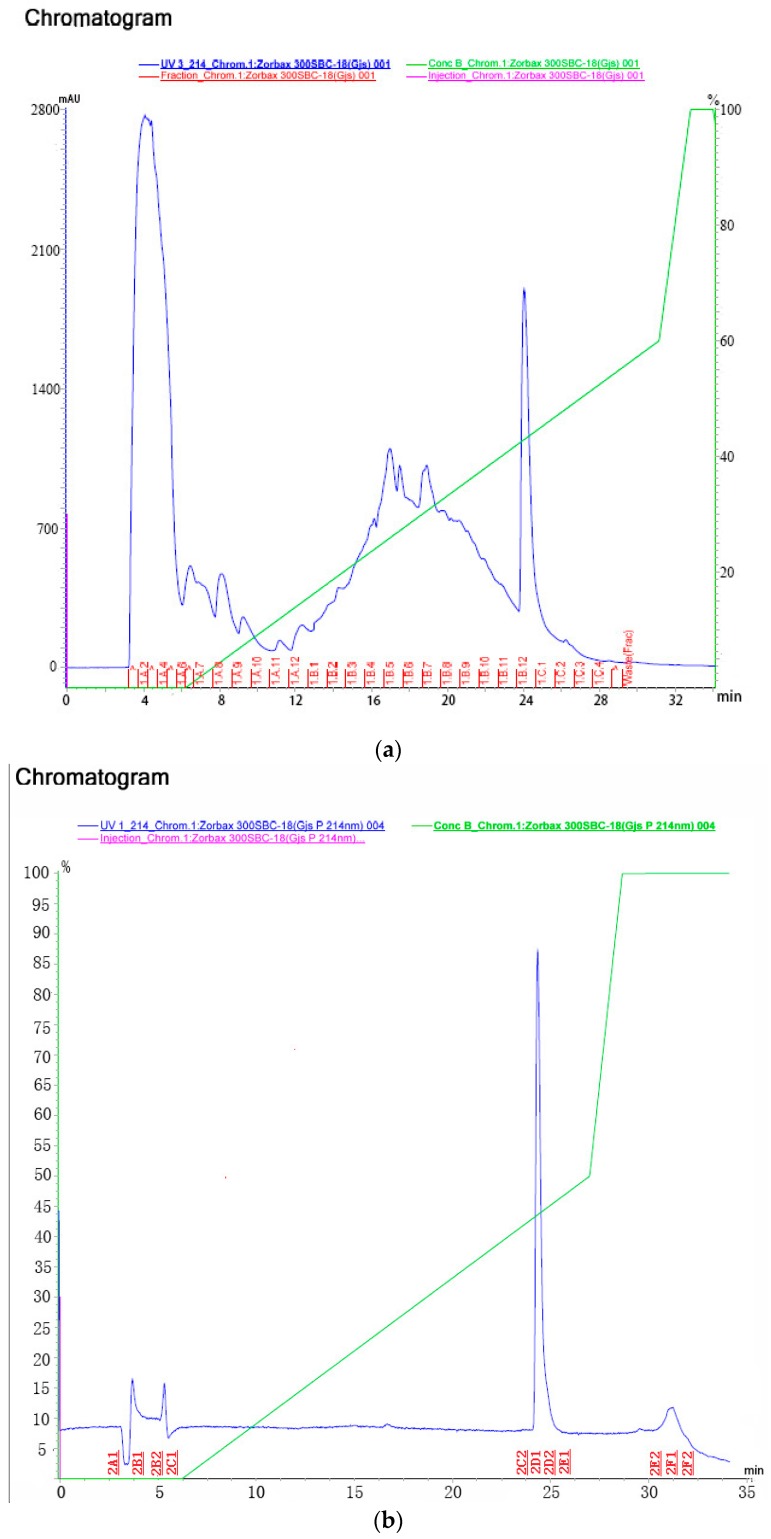
Purification by preparative HPLC in two steps: (**a**) eleven fractions in the first step; and (**b**) seven fractions in the second step.

To obtain the insecticidal active extract, 20 L fermentation medium of *S. laindensis* H008 was subjected to filtration, precipitation, centrifugation, concentration and gel filtration. With the bioassay-guided method, the insecticidal extract was purified by preparative HPLC in two steps. First, the extract was purified by HPLC as shown in Figure 3a. In the first step, eleven fractions were obtained and each fraction was investigated its insecticidal activity as shown in Table 3. The tenth fraction (No. 1B_12_-1C_1_) exhibited the highest insecticidal activity (95% mortality). Second, the fraction No. 1B_12_-1C_1_ was purified by HPLC as shown in Figure 3b. In the second step, seven fractions were obtained and each fraction’s insecticidal activity was also investigated as shown in Table 3. The fifth fraction (No. 2D_1_-2D_2_) exhibited the highest insecticidal activity (100% mortality). Then, the purified insecticidal peptide’s (fraction No. 2D_1_-2D_2_) purity (more than 97.12%) and concentration (0.7 mg/mL) was investigated. Finally, a total of 258.2 mg of the purified insecticidal peptide was ultimately obtained from 20 L of fermentation medium.

#### 2.3.2. Identification

**Figure 4:**
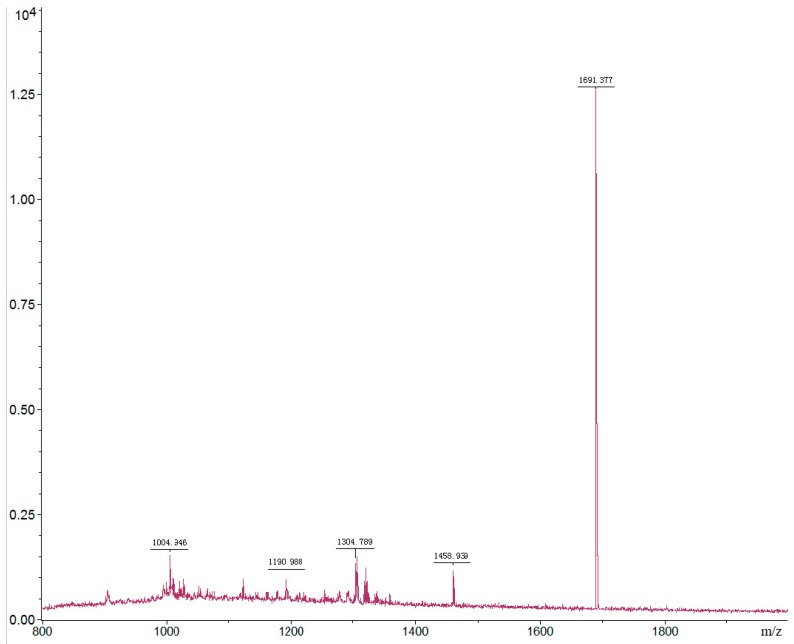
The TOF-MS analysis of peptide SLP1.

The purified insecticidal peptide (fraction No. 2D_1_-2D_2_) was analyzed by molecular mass determination and amino acid sequence analysis. The TOF-MS analysis showed that the molecular was 1691.377 Da (Figure 4). The analysis of amino acid sequence revealed that it was a 15 amino acid peptide (named SLP1). The amino acid sequence of SLP1 was determined to be S-E-P-A-Q-I-V-I-V-D-G-V-D-Y-W (Ser-Glu-Pro-Ala-Gln-Ile-Val-Ile-Val-Asp-Gly-Val-Asp-Tyr-Trp) as shown in Figure S1. As far as we know, the insecticidal peptide SLP1 is a novel peptide with the insecticidal activity against *L. erysimi*.

## 3. Discussion

According to the Kuster’s key for identification of *Streptomyces* spp. [14], *S. laindensis* H008 is only closed to *S. actuosus* by comparing 274 named taxa of *Streptomyces* spp. However, *S. actuosus* can utilize l-arabinose, l-rhamnose and raffinose [15], and *S. laindensis* H008 cannot utilize anyone of them. In addition, 16SrDNA of *S. laindensis* H008 is closed to *S. sampsonii* and *S. albus*. However, *S. laindensis* H008 belongs to the group of gray aerial mycelium and no distinctive reverse pigment, and both *S. sampsonii* and *S. albus* are not in this group [14]. Therefore, *S. laindensis* H008 is a novel species of genus *Streptomyces* based on its morphological and physiological traits and 16SrDNA analysis. Our result indicated that *S. laindensis* H008, a new species, produced a new insecticidal peptide SLP1 against *L. erysimi*. Therefore, *Streptomyces* spp. still deserve investigation of their new insecticidal peptide, although the *Streptomyces* species from soil have been widely researched [9,16,17,18]. 

The insecticidal peptide SLP1 exhibited similar insecticidal activity against *L. erysimi* (100% mortality at 700 mg/L; molecular weight: 1691.38) with the insecticidal compounds of low molecular weight such as the ethylene glycol diesters (LC_50_: 1320 to 11,500 mg/L) [19], the two derivatives of Harmine (LC_90_: 240.10 mg/L and 418.63 mg/L) [20] and three insecticidal alkaloids from *Cynanchum mongolicum* (LC_50_: 292.48 mg/L, 367.21 mg/L and 487.791 mg/L) [21]. Although the peptide SLP1 showed lower activity against *L. erysimi* than the protein of higher molecular weight from plants such as defensins (BjD and RiD) (LC_50_: 9.01 and 45.31 mg/L) [22] and *Allium sativum* leaf agglutinin (LC50: 20 mg/L) [23], the peptide SLP1 showed the good stability of pH and heat, and it is easier to be produced by a cost-effective and time-saving microbial fermentation method than cultivating plants [1,4]. It is because the species of *Streptomyces* has a shorter life cycle and a smaller genome than plants. It is easier to get a high-producing mutant of *Streptomyces* spp. than plants. The production of *Streptomyces* spp. metabolites is more easily improved by fermentation process optimization than optimizing plant cultivation.

Taken together, the insecticidal peptide SLP1 from *S. laindensis* H008 had the promising potential to be developed as a new biopesticide according to its stability of pH and heat, insecticidal activity and molecular character. The biosynthesis, active mechanism, structure–activity relationship and fermentation optimization of the insecticidal peptide SLP1 deserve to be further researched in future.

## 4. Materials and Methods

### 4.1. Microorganism

#### 4.1.1. Isolation

The soil samples were taken with an auger (diameter 2.5 cm) to a depth of 20 cm. Ten auger holdings were randomly collected from an agricultural field at the Institute of All-Russian Plant Conservation Biology, Russia. The soil dilution technique on glycerol-asparagine-salts agar medium. The strain was maintained on yeast extract-malt extract-dextrose (YMD) agar medium at 4 °C for further study [24].

#### 4.1.2. Morphological and Physiological Traits

Cultural characters of strain H008 were studied on International Streptomyces Project (ISP) and non-ISP media [25]. The micromorphology of the strain cultured on ISP medium 2 at 37 °C for 2 days was examined under scanning electron microscopy (model JOEL-JSM 5600, JOEL Limited, Akishima, Tokyo, Japan) at various magnifications [24,26]. Utilization of carbon sources was investigated [27].

#### 4.1.3. Molecular Biological Identification

Extraction of genomic DNA of the strain was performed according to the method described by Rainey et al. [28]. The 16SrRNA gene was amplified with primers 8-27f (5′-AGAGTTTGATCCTGGCTCAG-3′) and 1115-1100r (5′-AGGGTTGCGCTCGTTG-3′) [29,30,31,32]. The amplified DNA fragment was separated on 1% agarose gel, eluted and purified using the Qiaquick gel extraction kit (Qiagen, Hilden, Germany). The purified PCR product was sequenced using the Big-Dye terminator kit ABI 310 Genetic Analyzer (Applied Biosystems, Foster City, CA, USA). The phylogenetic position of the isolated strain (H008) was assessed by performing a nucleotide sequence database search using the BLAST (https://blast.ncbi.nlm.nih.gov/Blast.cgi). The sequence data of related species were retrieved from GenBank (http://www.ncbi.nlm.nih.gov/genbank/). The alignment and the phylogenetic tree were carried out by using Mega software (version 7.0 for windows) [33]. The number of Bootstrap Replications was set as 1000, and the 16S ribosomal RNA gene of *Kitasatospora setae* strain KM-6054 was used as an outgroup sequence.

#### 4.1.4. Fermentation

The bacterial stain was inoculated into a 500 mL Erlenmeyer flask containing 100 mL of seed medium composed of yeast extract (0.4%), malt extract (1.0%), dextrose (0.4%) and CaCO_3_ (0.2%) at pH 7.4. The inoculated flask was placed in rotary shaker (280 rpm) at 28 °C for 48 h. Seed culture (10% *v*/*v*) was transferred to the production medium consisting of sucrose (2%), tryptone (1%), K_2_HPO_4_ (0.05%), NaCl (0.05%) and FeSO_4_·7H_2_O (0.001%) at pH 6.5. The flask with the production medium was placed in a rotary shaker (280 rpm) at 30 °C for 96 h [24].

### 4.2. Insecticidal Activity

The aphids *L. erysimi* were used as a targeted aphid and they came from a laboratory colony originating from the Heilongjiang Province Academy of Sciences, Harbin, China. The wingless adults of the aphid *L. erysimi* were selected for the insecticidal test. The insecticidal activity of the culture filtrates, the fractions (in the purified process) and peptide SLP1 were tested by the leaf dipping method [20,21]. The leaf of cabbage with the *L. erysimi* was cut into the leaf discs. Then, the leaf disc with 20 (for the bioassay-guided purification) or 30 (for the stability) wingless adults of the *L. erysimi* was dipped into the culture filtrate or other solution (the fraction or peptide SLP1 solution) for 10 s, and the excess solution was immediately absorbed with a filter paper. Water was used as a control. The leaf disc with the *L. erysimi* in the petri dish were held at 25 ± 0.5 °C, light period of a 14/10-h light/dark cycle (LD), and 75%–85% relative humidity (RH). Each treatment was repeated three times. The mortality and the adjust mortality (with reference to the control) were calculated after 48 h.

### 4.3. Purification and Identification of Insecticidal Peptide

To the culture filtrate (20 L) of strain H008 was added acetone (50:50, *v/v*). The extract of peptide was obtained after the centrifugation, vacuum concentration and gel filtration (Sephadex G-15 column, 1.5 cm × 50 cm) [34]. With the bioassay-guided method, the extract of peptide was purified by preparative HPLC (Zorbax 300SB-C18 reverse-phase column, 4.6 mm × 100 mm, Agilent Technologies, Wilmington, DE, USA) (Solution A: 80% acetonitrile containing 0.05% TFA; Solution B: 0.065% TFA plus 2% acetonitrile) in two steps: First, the extract was purified by HPLC (0% Solution A for 6 min, followed by 0%–60% Solution A over 25 min, 1.5 mL/min, at 214 nm). Second, the fraction with the highest activity was also subjected to the second purification by HPLC (0% Solution A for 6 min, followed by 0%–50% Solution A over 20 min, 1.5 mL/min, at 214 nm) [35]. After purification of HPLC in two steps, the purity of the purified peptide with the insecticidal activity was investigated by HPLC. The concentration of the peptide was determined by the different concentrations of BSA solution.

The insecticidal peptide was analyzed using the Triple TOF 5600 TOF MS Analyzer (Applied 130 Biosystems, Concord, ON, Canada), and the data were acquired in the MS scanning mode with a scan range of 250–2000 (*m*/*z*) [34,36]. The insecticidal peptide was also submitted to an amino acid sequence analysis. The N-terminal amino acid sequences of the peptides were determined by Edman degradation carried out in a Shimadzu PSQ-23A protein sequencer (Shimadzu, Kyoto, Japan).

## 5. Conclusions

A novel insecticidal peptide SLP1 was isolated and characterized from a new species of *Streptomyces* (*S. laindensis* H008) against *L. erysimi* and it had the promising potential to be developed as a new biopesticide.

## Figures and Tables

**Table 1:** Cultural characteristics of strain H008.

Culture Media	Growth	Aerial Mycelium	Substrate Mycelium	Soluble Pigment
Tryptone	Abundant	Trace (Pale gray)	Brown	None
Yeast malt agar (ISP 2)	Abundant	Gray	Dark brown	None
Oatmeal agar (ISP 3)	Moderate	Gray	Colorless	None
Inorganic salt starch agar (ISP 4)	Moderate	Mouse gray	Colorless	None
Glycerol asparagine agar (ISP 5)	Poor	Trace (Pale gray)	Light brown	None
Peptone yeast extract iron agar (ISP 6)	Abundant	Gray	Brown	Yellow brown
Tyrosine agar (ISP 7)	Moderate	Gray	Colorless	None
Malt extract agar	Abundant	Gray	Dark brown	None
Maltose tryptone agar	Abundant	Gray	Brown	None
Nutrient agar	Moderate	Mouse gray	Colorless	None

**Table 2:** Physiological and biochemical characteristics of strain H008.

Test Items	Results	Utilization of Carbon Source	Results
Melanoid pigments	+	d-fructose	+
Production of H_2_S	+	d-glucose	+
Liquefaction of gelatin	+	d-galactose	+
Starch hydrolysis	+	d-mannitol	+
Coagulation of milk	−	d-xylose	+
Peptonization of milk	−	Inositol	+
Nitrate reduction	+	l-arabinose	−
Degradation of cellulose	−	l-rhamnose	−
Citrate utilization	+	Sucrose	+
Tolerance to NaCl	Up to 7%	Raffinose	−

Note: + means positive results; − means negative results.

**Table 3:** Insecticidal activity of each fraction purified by HPLC.

Step	Fraction No.	Mortality	Adjust Mortality
Step 1	1A_2_-1A_6_	10%	5.3%
	1A_7_	15%	10.5%
	1A_8_-1A_9_	10%	5.3%
	1A_10_	10%	5.3%
	1A_11_-1A_12_	15%	10.5%
	1B_1_-1B_3_	10%	5.3%
	1B_4_-1B_6_	5%	0
	1B_7_-1B_8_	15%	10.5%
	1B_9_-1B_11_	5%	0
	1B_12_-1C_1_	95%	94.7%
	1C_2_-1C_4_	10%	5.3%
	Control	5%	0
Step 2	2A_1_	10%	5.3%
	2B_1_	5%	0
	2B_2_	10%	5.3%
	2C_1_-2C_2_	5%	0
	2D_1_-2D_2_	100%	100%
	2E_1_-2E_2_	10%	5.3%
	2F_1_-2F_2_	15%	10.5%
	Control	5%	0

## Supplementary Materials

The following are available online at http://www.mdpi.com/1420-3049/21/8/1101/s1.
