# Supplementary material for: A Novel Insecticidal Peptide SLP1 Produced by Streptomyces laindensis H008 against Lipaphis erysimi
Source: Molecules. 2016 Aug 22;21(8):1101. doi: 10.3390/molecules21081101 (PMC6273262; doi:10.3390/molecules21081101)
Supplement: Supplementary file 1 [file molecules-21-01101-s001.pdf]

Supplementary Materials: A Novel Insecticidal Peptide SLP1 Produced by *Streptomyces laindensis* H008 against *Lipaphis erysimi*

Lijian Xu, Kangkang Liang, Bensha Duan, Mengdi Yu, Wei Meng, Qinggui Wang and Qiong Yu

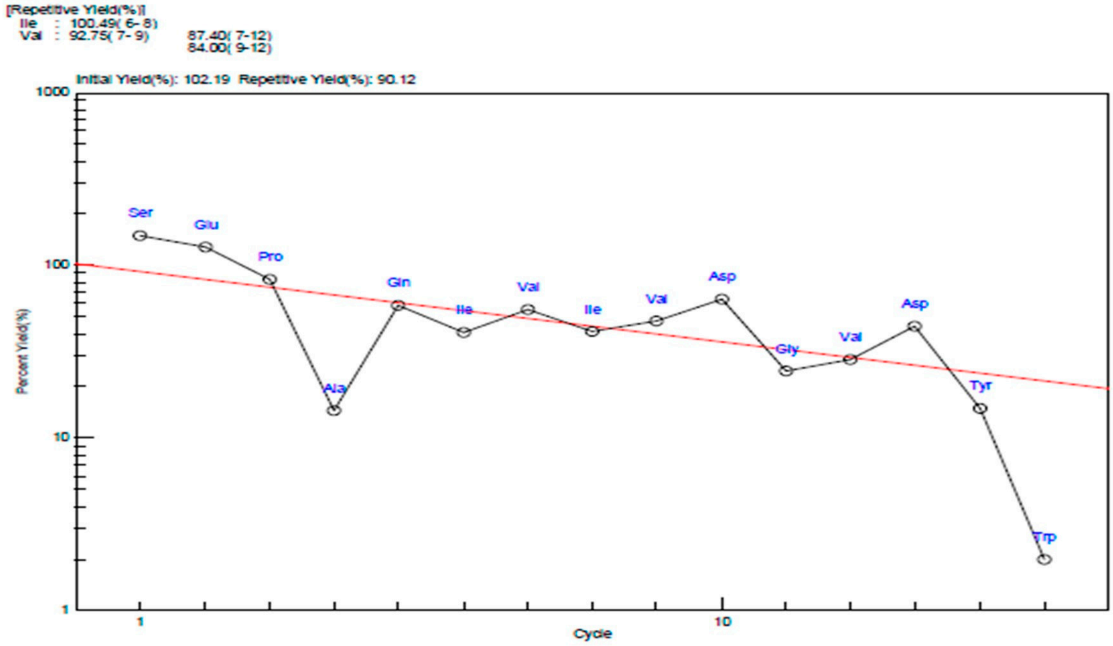

Figure S1. The amino acid sequence analysis of peptide SLP1.
